# High Factor VIII Levels and Recurrent Thromboembolism in Patients with and without Inflammatory Bowel Disease: A Retrospective Comparative Study

**DOI:** 10.1055/a-1827-7464

**Published:** 2022-06-13

**Authors:** G E. Eagle, Sam Schulman

**Affiliations:** 1 Faculty of Health Sciences, McMaster University, Hamilton, Ontario, Canada; 2 Department of Medicine, Thrombosis and Atherosclerosis Research Institute, McMaster University, Hamilton, Ontario, Canada; 3 Department of Obstetrics and Gynecology, I. M. Sechenov First Moscow State Medical University, Moscow, Russia

**Keywords:** inflammatory bowel disease, venous thromboembolism, factor VIII, oral anticoagulation, bleeding

## Abstract

**Background** The natural course of elevated factor VIII (FVIII) in patients with venous thromboembolism (VTE) and with or without inflammatory bowel disease (IBD) is not well described. Furthermore, the data on effectiveness and safety of extended anticoagulation in these patients are limited.

**Methods** We performed a retrospective chart review of all patients with VTE who had an elevated FVIII level (>1.5 IU/mL) during a period of 16 years. FVIII levels, duration of anticoagulation, recurrent thromboembolic events, and bleeding requiring hospitalization were captured and compared between patients with and without IBD.

**Results** Fourteen patients with IBD and 66 without IBD were followed for 8.0 years (standard deviation [SD] =  ±  3.5) and 5.6 years (SD =  ±  5.1), respectively. Among the 41 patients with repeat levels, FVIII remained elevated in most patients. None of the IBD patients had thromboembolic events or major bleeding during a mean of 5.6 years (SD =  ±  5.1) of anticoagulation. Three of five IBD patients who stopped anticoagulation had thromboembolic events at a median of 9 months after stopping, observed event rate of 12 per 100 patient-years. For the 66 non-IBD patients, the event rates of thromboembolism on and off anticoagulation were 1.6 and 7.2 per 100 patient-years, respectively, and of major bleeding on anticoagulation 0.8 per 100 patient-years.

**Conclusion** Elevated FVIII in patients with VTE is often a persistent risk factor. The cohort with VTE and elevated FVIII that we analyzed appeared to have a favorable benefit/risk ratio of extended anticoagulation.

## Introduction

Elevated levels of several coagulation factors have been associated with increased risk for venous thromboembolism (VTE), and with the strongest association for factor VIII (FVIII) and von Willebrand factor. 1 Another study showed that in multivariable analysis, only FVIII activity (and not the von Willebrand factor or non-O blood group) was an independent risk factor. 2 High FVIII is also independent of elevated D-dimer as risk factor for recurrent VTE. 3 In a study of 360 patients with VTE, the adjusted risk for recurrence was 6.7 times higher among those with FVIII levels above the 90th percentile, that is, ≥2.35 IU/mL compared with those with lower levels. 4 Although this has often been used as cut-off for the risk assessment, Wells et al proposed based on another study with 300 patients, a higher cut-off at 2.70 IU/mL. 5 The risk of VTE recurrence is, however, proportional to FVIII levels, with relative risk of 1.08 (95% confidence interval [CI]: 1.04–1.12) for every 0.1 IU/mL increase in FVIII, 4 and in patients with additional risk factors, even mildly increased FVIII, may contribute to a clinically important risk of recurrence.

Elevated FVIII is associated with older age, to African-American ethnicity (probably due to lower prevalence of blood group O), and to acute phase response. 6 Familial clustering has been observed but so far, only one genetic defect, a partial *F8* gene duplication in FVIII Padua has been linked with very high plasma levels of FVIII. 7

Elevated FVIII levels are commonly found in patients with inflammatory bowel disease (IBD). 8 9 10 11 12 A study in pediatric patients diagnosed with Crohn's disease found a positive association between FVIII levels, disease activity, and other markers of inflammation such as erythrocyte sedimentation rate, serum orosomucoid, albumin, and C-reactive protein. 13 Similar correlations of elevated FVIII with disease activity have been made for ulcerative colitis. 10 On the other hand, in the Leiden Thrombophilia Study, high FVIII levels independently increased the risk of VTE after adjustment for C-reactive protein levels and thus is not believed to be caused by acute phase reactions. 14

Far from all patients with VTE and high FVIII have IBD, and often no comorbidity is identified. We aimed in this study to compare outcomes in patients with VTE and elevated FVIII with or without IBD with regard to trajectories of FVIII, recurrence of thromboembolic events, and effectiveness of anticoagulant treatment.

## Methods

This was a retrospective chart review of patients assessed by the Thrombosis Service at the Hamilton General Hospital for VTE and who had thrombophilia work-up, including FVIII level, during the period of September 1, 2004, to August 31, 2020. The patients were identified by searching our records for “FVIII” and excluding those with normal or low levels. Patients less than 18 years of age or with known cancer were also excluded. The study was approved by our Research Ethics Board as a retrospective chart review without need for informed consent. The study was also a BHSc (Bachelor of Health Science) project course for the primary author. No funding was obtained for the study.

### Study Procedures

We reviewed the records of the Thrombosis Service, as well as the electronic records of Hamilton Health Sciences, which include all the sites of this hospital organization. Data on demography (age, sex, and weight), the thromboembolic event leading to the investigation (date and anatomic location), previous VTE(s), comorbidities (IBD, cardiovascular diseases, diabetes, pulmonary, kidney, or liver disease and smoking habit), anticoagulant management (type, duration, and concomitant antiplatelet agent), FVIII levels, recurrent thromboembolic events, deaths, hospitalizations, and, for IBD patients, information on disease flares were collected. Information of medications for IBD was available for the time of the visits to the hospital but not reliable regarding continuity.

The starting time point for the follow-up was the first FVIII test. The sampling for this was at least 3 months after the most recent thromboembolic event. In almost all patients, this was while still on anticoagulation. The FVIII activity was measured with a clotting assay using FVIII Deficient Plasma (Affinity Biologicals, Ancaster, Ontario, Canada) and activated partial thromboplastin time reagent (Siemens Dade Actin FS, Siemens, Marburg, Germany) on a STAR/STAR Evolution analyzer Diagnostica Stago, Asnieres sur Seine, France). Any repeat FVIII tests were performed at the discretion of the treating physician. Elevated FVIII was above 1.5 IU/mL. We further stratified according to the first FVIII level (>1.50 to 2.34 vs. >2.34 IU/mL).

### Outcomes

Laboratory outcomes were the trajectory of FVIII levels and shifts in strata. Clinical outcomes were recurrent thromboembolic events during anticoagulation or after first discontinuation. Temporary interruption of anticoagulation for surgery or for management of bleeding was not considered discontinuation. Thromboembolic events could be venous or arterial and had to be objectively verified. Such events could be extremity and nonextremity deep vein thrombosis, ischemic stroke, and systemic embolism. Superficial thrombophlebitis was not considered an outcome. The only major bleeds that could be reliably captured were those requiring hospitalization.

### Statistical Analysis

Due to the limited number of patients in the IBD group and a small number of recurrent thromboembolic events, the statistics are mainly descriptive with mean and standard deviation (SD) or median and interquartile range (IQR) for normal or skewed distribution, respectively, as verified with Shapiro–Wilk test. Comparisons of characteristics between the IBD- and the non-IBD groups are analyzed with Fisher's exact test and comparison of the FVIII level at baseline is with Mann–Whitney *U* -test. A *p* -value of <0.05 was considered statistically significant.

## Results

Out of 218 patients tested, we identified 80 patients (37%) with elevated FVIII, 14 with IBD and 66 without IBD. Of the former, six had been diagnosed with Crohn's disease and eight with ulcerative colitis with a mean duration before the first FVIII test of 11 years (SD =  ±  8). Among the 66 patients without IBD, the majority (83%) had no comorbidity that would explain high FVIII, 5 had osteoarthritis, and one each had polyarthritis, temporal arteritis, undifferentiated connective tissue disease, sarcoidosis, chronic pancreatitis, and recurrent infections. The baseline characteristics of the two groups are shown in Table 1 . Patients without IBD had a higher body weight than those with IBD, and 10 among the former weighed more than 100 kg. Five of the 14 IBD patients (36%) were on oral steroids and one was using steroid enemas at the time of the first FVIII level.

**Table 1:** Baseline characteristics of patients with high FVIII level

Variable	IBD patients *n* (%)/mean ± SD	Non-IBD patients *n* (%)/mean ± SD	*p* -Value
*n*	14	66	
Age (y)	47.9 ± 11.3	50.0 ± 14.9	0.60
Sex (female)	10 (71)	31 (47)	0.14
Weight (kg)	70.7 ± 15.7	88.4 ± 22.1	0.024
Index thrombotic event a			
Deep vein thrombosis	13	27	
Pulmonary embolism	1	25	
Splanchnic vein	0	9	
Cerebral/sinus vein	1	9	
Arterial thrombosis	2 b	6 b	
Previous history of VTE	5 (36)	20 (30)	0.75
Comorbidities			
Thrombophilic defect	1 (7)	18 (27)	0.17
Cardiovascular disease	5 (36)	24 (36)	1.0
Hypertension	4 (29)	18 (27)	1.0
Diabetes	1 (7)	7 (11)	1.0
Chronic kidney disease	0	0	

Abbreviations: IBD, inflammatory bowel disease; F, factor; SD, standard deviation; VTE, venous thromboembolism.

a Patients could have had thrombotic events in more than one location. Index thrombotic event is the VTE within a year before the first FVIII test.

b Although these patients were referred for an arterial event, both IBD patients and 4 of the 6 non-IBD patients had definitive VTE events in their history.

### Factor VIII Levels and Trajectories

**Fig. 1:**
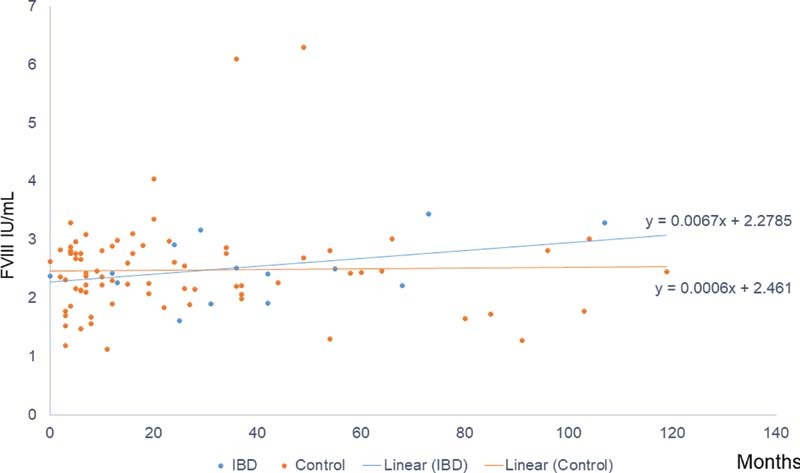
FVIII levels over time. Time point 0 shows the median of the first FVIII tests. F, factor; IBD, inflammatory bowel disease. Controls are the patients with high FVIII and no IBD.

The median FVIII level of the first test was 2.41 IU/mL (IQR: 2.08–2.50) for the patients with IBD of whom seven (50%) had a level of 2.35 IU/mL or higher. For the non-IBD patients, the median level was slightly higher at 2.63 IU/mL (IQR: 2.39–2.88 *p* = 0.058), and 54 patients (82%) showed a level of ≥2.35 IU/mL ( *p* = 0.018). Seven patients, all in the non-IBD group, with initial FVIII <2.35 IU mL had levels above that limit during the follow-up. Table 2 shows changes in FVIII levels from the first to the second test. Many of the patients, who had a lower FVIII level on the second test, rebounded on further tests. Only three patients had FVIII levels that at any point decreased into the normal range; for two of those confirmed with repeated test after at least 6 months. The FVIII trajectories overall are shown in Fig. 1 . There is essentially a flat regression line for both groups.

**Table 2:** Follow-up of patients with high FVIII

Variable	IBD patients *n* (%)/mean ± SD	Non-IBD-patients *n* (%)/mean ± SD	*p* -Value
*n*	14	66	
Duration of follow-up (y) a	8.0 ± 3.5	7.4 ± 4.3	0.63
Oral anticoagulation (y) a	5.6 ± 5.1	5.5 ± 4.5	0.94
Concomitant antiplatelet treatment	2 (14)	6 (9)	0.62
Repeat FVIII performed	4	37	
Increase of FVIII from <2.35 to ≥2.35 (IU/mL)	0	7 (19)	
Decrease of FVIII from ≥2.35 (IU/mL)	2 (50)	14 (38)	
To 1.5–2.34 IU/mL b	2	12	
To <1.50 IU/mL b	0	2	

Abbreviations: IBD, inflammatory bowel disease; F, factor; SD, standard deviation.

a Counting from the first FVIII test as starting point and until last contact registered for follow-up, or end of treatment or last contact for duration of anticoagulation, whatever came first.

b Percent of those with repeat FVIII tests.

### Clinical Follow-up

**Fig. 2:**
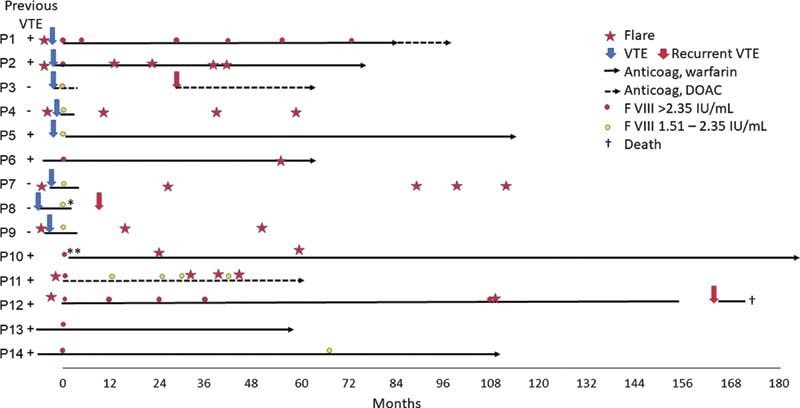
Overview of the events in patients with inflammatory bowel disease. Time point 0 is at the first FVIII test. Recurrent VTE events during the study are red-filled arrows. The blue-filled arrows are VTEs a few months before the first FVIII test. Those without this arrow have the + sign for “Previous VTE.” DOAC, direct oral anticoagulant; F, factor; VTE, venous thromboembolism.

During a mean follow-up of 8.0 years (SD =  ±  3.5) in the IBD group and a mean duration of anticoagulation (counted from the first FVIII test) of 5.6 years (SD =  ±  5.1), three patients (21%) experienced recurrent VTE, none of which happened on anticoagulant treatment ( Fig. 2 ). Thus, there were no thromboembolic events during 79 patient-years on anticoagulation (or with 95% confidence interval [CI]: 0–23% during a mean of 5.6 years on anticoagulation). Eight of the patients continued to have flares of the IBD with one to four episodes documented. The recurrent events were, however, not associated with such flares. The mean follow-up after discontinuation of anticoagulation (six patients) was 4.1 years (SD =  ±  3.3). Three of the five patients that discontinued anticoagulation had VTEs, two deep vein thrombosis, and one pulmonary embolism at 4, 9, and 28 months (median 9 months) after stopping, respectively. Therefore, we observed three recurrences during a total of 24.6 years off anticoagulation which translates to 12 events per 100 patient-years (or with 95% CI: 12–88% during a mean of 4.1 years).

During a mean follow-up of 7.4 years (SD =  ±  4.3) in the non-IBD group, 13 patients (20%) had new thromboembolic events ( Table 3 ). The mean duration of anticoagulation was 5.5 years (SD =  ±  4.5) and six of the thromboembolic events occurred on anticoagulant treatment, but four of those occurred while being subtherapeutic. Seven of the 32 patients (22%) who discontinued anticoagulation were diagnosed with new thromboembolic events a median of 4 months (IQR: 2–13.5) after stopping. The follow-up after discontinuation of anticoagulation was very short in this subgroup, median of 0.1 years (IQR: 0–1.6). The thromboembolic events in the non-IBD patients consisted of deep vein thrombosis ( *n* = 6), pulmonary embolism ( *n* = 2), deep vein thrombosis and pulmonary embolism ( *n* = 2), rethrombosis of portal vein ( *n* = 1), and ischemic stroke ( *n* = 2). The event rate on anticoagulation was 1.6 per 100 patient-years (or with 95% CI: 3–19% during a mean of 5.5 years), and off anticoagulation, it was 7.2 per 100 patient-years (or with 95% CI: 9–40% during a median follow-up of 0.3 years).

**Table 3:** Thromboembolic events during follow-up

Variable	IBD patients *n* (%)/mean ± SD/median (IQR)	Non-IBD-patients *n* (%)/mean ± SD/median (IQR)	*p* -Value
*n*	14	66
Recurrent thromboembolic events	3 (21)	13 (20)	1.0
On anticoagulation	0	6	
Duration of anticoagulation until event (y) a	NA	7.2 ± 4.8	
After stopping anticoagulation	2	7	
Duration of anticoagulation until stopping (y) a		4.8 ± 5.0	
Interval from stopping anticoagulation (y)	0.8 (0.2–1.5)	0.3 (0.2–1.5)	

Abbreviations: IBD, inflammatory bowel disease; IQR, interquartile range; SD, standard deviation.

a Using the first factor (F) factor VIII test as starting point.

Major bleeds on anticoagulation and with hospitalization were not recorded during follow-up in any of the IBD patients. It was recorded in three of the patients without IBD (intracranial hemorrhage, trauma-associated subarachnoid hemorrhage, and gastrointestinal hemorrhage associated with cancer that was diagnosed during the follow-up), corresponding to an event rate of 0.8 per 100 patient-years on anticoagulation. During the follow-up, cancer was diagnosed in five patients (8%) in the group without IBD.

In the two groups combined, 19 had initially a moderate elevation of FVIII (1.5–2.34 IU/mL) and 3 of those (16%; 95% CI: 3–40%) suffered recurrent thromboembolic events whereas 51 had a more pronounced elevation (≥2.35 IU/mL) and 13 of those (25%; 95% CI: 14–40%) had recurrent thromboembolic events ( *p* = 0.53).

## Discussion

In this retrospective analysis of patients with a history of VTE and with an elevated FVIII level, we found that these levels rarely normalized over time and that in both groups the trajectories stayed flat over many years. The risk of new thromboembolic events on anticoagulation was low, 0 per 79 patient-years in the IBD group and 1.6 per 100 patient-years in the non-IBD-group. Conversely, the risk of such events was quite high after discontinuation of anticoagulation, occurring in 60 and 22% of patients with IBD and without IBD, respectively, after a median of 9 and 4 months, respectively, and resulting in an approximate event rate of 12 per 100 patient-years and 7.2 per 100 patient-years, respectively. Although the event rate in the IBD group was numerically higher, the difference was not statistically significant. In view of the short duration of follow-up after discontinuation of anticoagulation in the non-IBD group, the true event rate is most likely higher. The risk of new thromboembolic events was numerically higher in patients with pronounced elevation of FVIII compared with those with moderate elevation but again without a statistically significant difference. The risk of bleeding on anticoagulation was low in both groups.

In the group without IBD, we only found a plausible etiology, related to chronic inflammation, in 17% of the cases. We did not have any patients of African American ethnicity and samples were obtained several months after the most recent thromboembolic event to avoid acute phase effect. Occult cancer might have been present at the time of sampling in another 8% with elevated FVIII, yet in 75% of the cases, we did not detect any underlying pathology. The patients without IBD had a higher body weight than those with IBD, and obesity is associated with chronic inflammation 15 and also with hemostatic abnormalities including elevated FVIII. 16 Furthermore, there seems to be an association between steroid therapy which was used by one-third of the patients with IBD, elevated FVIII levels, and thrombosis. 17

The risk of recurrent thromboembolic events on anticoagulation in our two groups was similar to the low risk reported in a meta-analysis of 26 studies that included patients with first unprovoked VTE and with extended oral anticoagulant treatment, namely, 1.41 per 100 patient-years. 18 Furthermore, the risk of recurrent events after discontinuation of anticoagulation was of the same magnitude as the 10.3 events per 100 patient-years (during the first year) in another meta-analysis of 18 studies with patients treated after first unprovoked VTE. 19 The guidelines of American Society of Hematology on VTE suggest indefinite duration of anticoagulation after unprovoked VTE, as well as after VTE “provoked by a chronic persistent risk factor,” 20 which include both IBD and elevated FVIII. In the CHEST guidelines, Update 2 provides a stronger statement and “recommends offering extended-phase anticoagulation” for VTE that is “unprovoked or provoked by persistent risk factor.” 21 Coremans et al recently published algorithms for anticoagulant management of VTE in IBD and gave a weak recommendation for indefinite duration of anticoagulation in case of acute VTE while in remission and a strong recommendation for indefinite treatment in case of recurrent VTE. 22 These algorithms do not discuss elevated FVIII as a risk factor. The European Crohn's and Colitis Organization guideline states that indefinite duration of anticoagulation should be discussed with patients with IBD and VTE without additional risk factor but also recommends that treatment of patients with IBD should not differ from protocols for other patients. 23

Investigation for thrombophilia is typically focusing on congenital defects and antiphospholipid syndrome but does not routinely include FVIII. Since elevated FVIII has been identified as a risk factor, independent of D-dimer for recurrent VTE, 3 we believe that it should be included in the investigation. The cut-off at the 90th percentile (≥2.35 IU/mL) is obviously somewhat artificial, since the risk of recurrence increases gradually with the FVIII level, 4 and thus moderate FVIII elevation also poses some additional risk, as seen in our analysis as well.

The decision on long-term anticoagulation must always take the risk of bleeding into account. For patients with ulcerative colitis the concern is that a flare will manifest with severe bleeding in case of concomitant anticoagulation. The only major gastrointestinal bleed recorded in our study was in a non-IBD patient with gastrointestinal malignancy, and overall the rate of major bleeding requiring hospitalization was low. A retrospective study on 107 patients with IBD reported a higher event rate of major bleeding during anticoagulation, that is, 2.6 per 100 patient-years, but it still concluded that this risk was probably outweighed by the high risk of recurrent VTE. 24

## Limitations

Our study has several limitations, first that it is a retrospective chart review and the patients who were referred to our service for investigation and advice were most likely selected. Second, the number of patients, particularly those with IBD, is small. Third, patients with elevated FVIII were not consistently tested again. We believe, however, that the long duration of clinical follow-up of the patients with IBD and of patients in both groups while on anticoagulation is a strength.

## Conclusion

In conclusion, patients with a history of VTE and with elevated FVIII, tested several months after the last episode to avoid acute phase effect, appeared to have a relatively high risk of recurrent thromboembolic events, and similar to those with unprovoked VTE. The elevation of FVIII tended to be chronic. These patients also seemed to have a favorable benefit/risk ratio of extended anticoagulation. These observations were made both in patient with and without IBD. It is probably useful to routinely include FVIII in the thrombophilia workup when it is performed to support decisions on duration of anticoagulation.
